# Diagnostics of Infections Produced by the Plant Viruses TMV, TEV, and PVX with CRISPR-Cas12 and CRISPR-Cas13

**DOI:** 10.1021/acssynbio.2c00090

**Published:** 2022-07-06

**Authors:** María-Carmen Marqués, Javier Sánchez-Vicente, Raúl Ruiz, Roser Montagud-Martínez, Rosa Márquez-Costa, Gustavo Gómez, Alberto Carbonell, José-Antonio Daròs, Guillermo Rodrigo

**Affiliations:** † Institute for Integrative Systems Biology (I2SysBio), CSIC—Universitat de València, Paterna 46980, Spain; ‡ Instituto de Biología Molecular y Celular de Plantas, CSIC—Universitat Politècnica de València, València 46022, Spain

**Keywords:** nucleic acid detection, CRISPR diagnostics, multiplexed diagnostics, plant virus

## Abstract

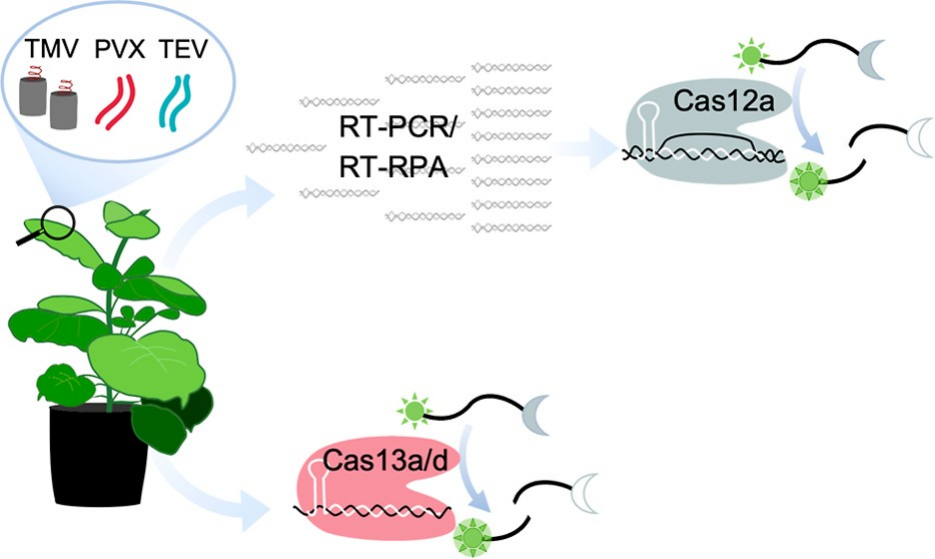

Viral infections in plants threaten food security. Thus, simple and effective methods for virus detection are required to adopt early measures that can prevent virus spread. However, current methods based on the amplification of the viral genome by polymerase chain reaction (PCR) require laboratory conditions. Here, we exploited the CRISPR-Cas12a and CRISPR-Cas13a/d systems to detect three RNA viruses, namely, *Tobacco mosaic virus*, *Tobacco etch virus*, and *Potato virus X*, in *Nicotiana benthamiana* plants. We applied the CRISPR-Cas12a system to detect viral DNA amplicons generated by PCR or isothermal amplification, and we also performed a multiplexed detection in plants with mixed infections. In addition, we adapted the detection system to bypass the costly RNA purification step and to get a visible readout with lateral flow strips. Finally, we applied the CRISPR-Cas13a/d system to directly detect viral RNA, thereby avoiding the necessity of a preamplification step and obtaining a readout that scales with the viral load. These approaches allow for the performance of viral diagnostics within half an hour of leaf harvest and are hence potentially relevant for field-deployable applications.

## Introduction

Infections in plants and animals represent a global threat to food security and public health, with viruses being a major class among pathogens.^1^ In plants, RNA viruses encompass most of the diversity found in nature^2^ and account for substantial losses in crop yields worldwide.^3^ Although plant breeding and transformation-based strategies can lead to genetic resistance to infection, prophylaxis programs focused on restraining virus spread are valuable strategies to control virus-induced diseases *via* quarantine periods, certification, control of infected reservoirs, and control of transmission vectors.^4^ Therefore, the availability of rapid, accurate, and cost-effective diagnostic methods for field-deployable detection of plant viruses is crucial to control viral diseases and preserve crop yields. This is especially relevant in the current scenario of human population pressure, intensive farming, and climate change in which production is under constant stress and the sustainability of the system is compromised.^5^ Hence, advancements in the development and application of novel field-deployable diagnostic methods for plant viruses are definitively required.

Current viral diagnostics typically include a procedure to amplify a viral genome fragment, such as the polymerase chain reaction (PCR) and its derivative approaches [e.g., reverse transcription followed by quantitative PCR (RT-qPCR)], or to detect viral proteins, such as the enzyme-linked immunosorbent assay.^6^ These methods require sophisticated equipment, infrastructure, and skilled technical staff. Furthermore, isothermal amplification-based methods such as loop-mediated isothermal amplification (LAMP) or recombinase polymerase amplification (RPA) have been applied to detect several plant viruses.^7,8^ Although these techniques lack high specificity, they are fast, sensitive, and require minimal sample preparation and equipment; therefore, they are useful in field-based scenarios. To solve the specificity issue, systems based on clustered regularly interspaced short palindromic repeats (CRISPR) and CRISPR-associated (Cas) proteins have been repurposed in recent years for diagnostic applications in combination with isothermal amplification methods^9,10^ (see ref^11^ for a review).

CRISPR-Cas systems are based on RNA-guided endonucleases and are derived from an effective immune system protecting bacteria and archaea against invading nucleic acids.^12^ These systems have been used in eukaryotes for genome editing^13^ and gene regulation,^14^ but have also been used for *in vitro* diagnostics thanks to their exquisite sequence specificity and their nonspecific collateral cleavage activity.^11^ In plants, the DNA-targeting, RNA-guided nuclease Cas12a, which can cleave in trans single-stranded DNA (ssDNA) molecules, was used very recently to detect RNA viruses (and even viroids) in combination with RT-RPA^15,16^ and also RT-PCR.^17^ However, there are still important issues to be addressed, such as how the system performs in conditions of complex mixed infections, if an RNA purification step is required, or if the viral RNA can be detected without amplification by means of a Cas ribonuclease.

In this work, we report the rapid, specific, and simple detection of plant RNA viruses in single and mixed infections in *Nicotiana benthamiana* (*N. benthamiana*) plants. For this, we followed a Cas12a-based detection procedure after viral amplification by RT-PCR or RT-RPA. We chose the Cas12a from *Lachnospiraceae bacterium* (*L. bacterium*) for our assays, as this ∼150 kDa nuclease has been shown very efficient to target DNA molecules, displays a marked collateral cleavage activity upon targeting, and maintains good performance at low temperatures. Moreover, we performed the detection and visualization from plant leaves with no RNA purification and using lateral flow strips. We further demonstrated that a Cas13a/d-based detection of plant virus RNA without amplification is possible, which in turn allowed the quantification of the viral titers in the plant extracts (Cas13a/d is an RNA-targeting, RNA-guided nuclease^12^). For our assays, we chose the Cas13a from *Leptotrichia buccalis* due to the superior trans-cleavage activity of this ∼130 kDa nuclease and the absence of target-flanking sequence requirements.^18^ We also considered the Cas13d from *Ruminococcus flavefaciens* due to its more compact size (almost 300 amino acid residues of difference with respect to other Cas13s, resulting in a ∼110 kDa nuclease).^19^

To perform this study, we considered *Tobacco mosaic virus* (TMV), *Tobacco etch virus* (TEV), and *Potato virus X* (PVX), three (+)-strand RNA viruses that are among the most important plant viruses based on scientific and economic criteria.^3^ PVX belongs to the family *Alphaflexiviridae* (genus *Potexvirus*) and causes disease (stunting) mainly in potato plants.^20^ The PVX genome consists of a ∼6.4 kb RNA that is capped at the 5′ end and polyadenylated at the 3′ end. Five viral proteins are expressed from the genomic and three subgenomic RNAs.^21^ TMV is a member of the family *Virgaviridae* (genus *Tobamovirus*), has a wide host range, and induces mosaic-like mottling and discoloration on the leaves.^22^ The TMV RNA genome is also ∼6.4 kb long but contains cap and tRNA-like structures at the 5′ and 3′ ends, respectively, and encodes four proteins. Expression is based on the translation of the genomic and two subgenomic RNAs.^23^ TEV belongs to the family *Potyviridae* (genus *Potyvirus*) and infects several *Solanaceae* species (e.g., pepper, tomato, and tobacco), inducing vein clearing, mottling, and necrotic lines or etching. The TEV genome consists of a ∼9.5 kb RNA with a viral protein genome-linked (VPg) and a poly(A) tail at the 5′ and 3′ ends respectively. Potyvirus expression strategy is mainly based on producing a large polyprotein that is processed by three virus-encoded proteases, leading to eleven proteins.^24^

## Results

### Detection of Plant Viruses with CRISPR-Cas12a

**Figure 1:**
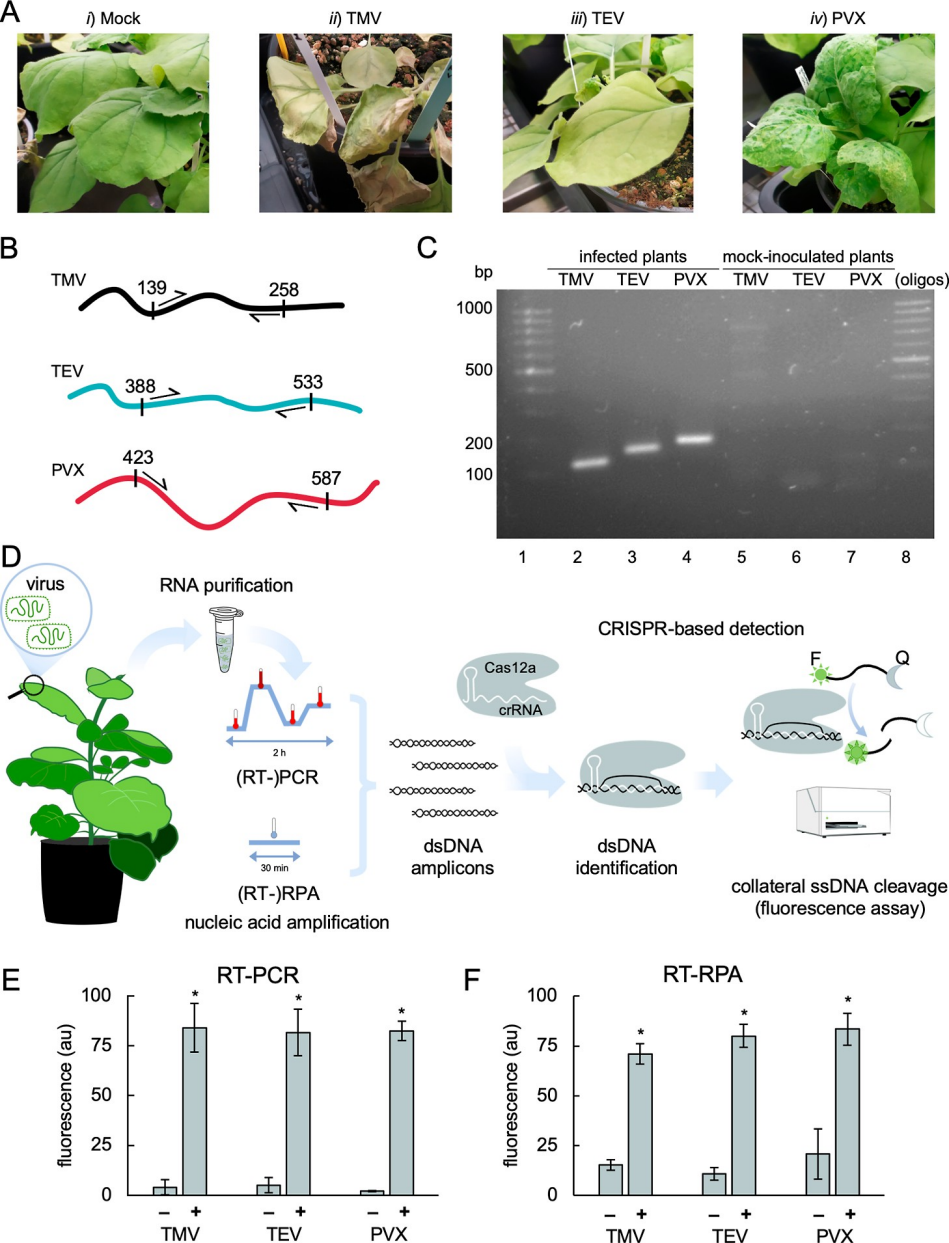
Plant virus detection with CRISPR-Cas12a. (A) Representative images of *N. benthamiana* plants infected with different plant RNA viruses at 7 dpi. From left to right: (i) mock-inoculated plants displayed no disease symptoms, (ii) TMV-induced necrosis, (iii) TEV-induced yellowing in the plant, and (iv) PVX-induced severe mosaic. (B) Viral genome schemes targeted by the PCR primers for amplification, with position numbers relative to the start codon of each coat protein. (C) Electrophoretic assay of RT-PCR performed from total RNA preparations of infected plants. (D) Schematic representation of CRISPR-Cas12a-based detection of plant viruses by a fluorescence readout. Two amplification methods were used: RT-PCR and isothermal RT-RPA. The ssDNA probe is labeled with a fluorophore (F, sun icon) and a quencher (Q, moon icon). (E,F) End-point fluorescence at 1 h measured in samples amplified by either RT-PCR (E) or RT-RPA (F) of plant material infected with TMV, TEV, or PVX (+) or mock-inoculated material (−). Error bars represent standard deviations (*n* = 4, two different amplifications and two CRISPR-Cas12a reactions per amplification). Statistical significance (Welch’s *t*-test, two-tailed *P* < 0.05) of higher fluorescence with respect to mock-inoculated plants (*). In (E), fold change (± standard error) was 21.49 ± 10.53 for TMV, 16.15 ± 6.15 for TEV, and 40.47 ± 3.42 for PVX. In (F), fold change (± standard error) was 4.67 ± 0.43 for TMV, 7.49 ± 1.11 for TEV, and 4.01 ± 1.23 for PVX.

RNA was purified from tissues collected from *N. benthamiana* plants infected with each virus, namely TMV, TEV, and PVX (Figure 1A). We designed a set of oligonucleotides to amplify a specific genomic region in each of the studied viruses, each with a different size (Figure 1B). We restricted the design to the region coding for the coat protein, which is located at the 3′ end of each viral genome, to ensure that almost all viral RNAs (complete genome and eventual subgenomic RNAs) may be detected (see the different virus genome architectures in Figure S1). The specific amplification of the different virus-derived double-stranded DNAs (dsDNAs) by RT-PCR was confirmed by gel electrophoresis (Figure 1C). Three Cas12a-dependent CRISPR RNAs (crRNAs) were designed to target the amplicons (one crRNA per virus) carrying a protospacer adjacent motif (PAM) for Cas12a recognition, trying to avoid cross-interaction with the host genome. All crRNAs were generated by *in vitro* transcription, Cas12a was a commercial preparation, and the ribonucleoproteins were assembled *in vitro* before the detection reactions.

We ran CRISPR-Cas12a reactions to detect the plant viruses using a fluorogenic ssDNA molecule as a probe (Figure 1D). We adopted two different approaches to generate the virus-derived DNA amplicons: RT-PCR and RT-RPA. We found that both approaches give similar results, offering good detectability (Figure 1E,F). As a control approach, viruses were also detected by RT-qPCR (Figure S2), the gold standard in diagnostics. In these assays, the primers used to amplify TEV did not produce detectable material from noninfected plants. To investigate the limit of detection, we prepared a series of diluted samples (up to a factor of 10^10^ from the original material, simulating scenarios in which the virus is present at lower concentrations due to a shorter collection time or poor accumulation). Figure S3 displays the results (in this case, amplified by RT-PCR). Furthermore, we tested the ability of the method to detect a plant virus in another organism. For that, we used purified RNA samples obtained from infected tissues of *Arabidopsis thaliana* (*A. thaliana*) plants with TEV,^25^ in which the virus was successfully detected with CRISPR-Cas12a (Figure S4).

Importantly, we found that RT-RPA allows amplification of the viral genome in just 30 min (in contrast to the 2 h required for RT-PCR); but it generates more background signal, arguably due to spurious amplification. Moreover, Figure S5 shows the kinetic curves of ssDNA cleavage by the active crRNA-Cas12a-dsDNA complex, revealing that just 5 min of the CRISPR-Cas12a reaction is enough to differentiate an infected plant from a mock-inoculated control by fluorescence. Notably, the entire diagnostic test from purified RNA samples only took 35 min when RT-RPA was used.

### Multiplexed Detection of Plant Viruses with CRISPR-Cas12a

**Figure 2:**
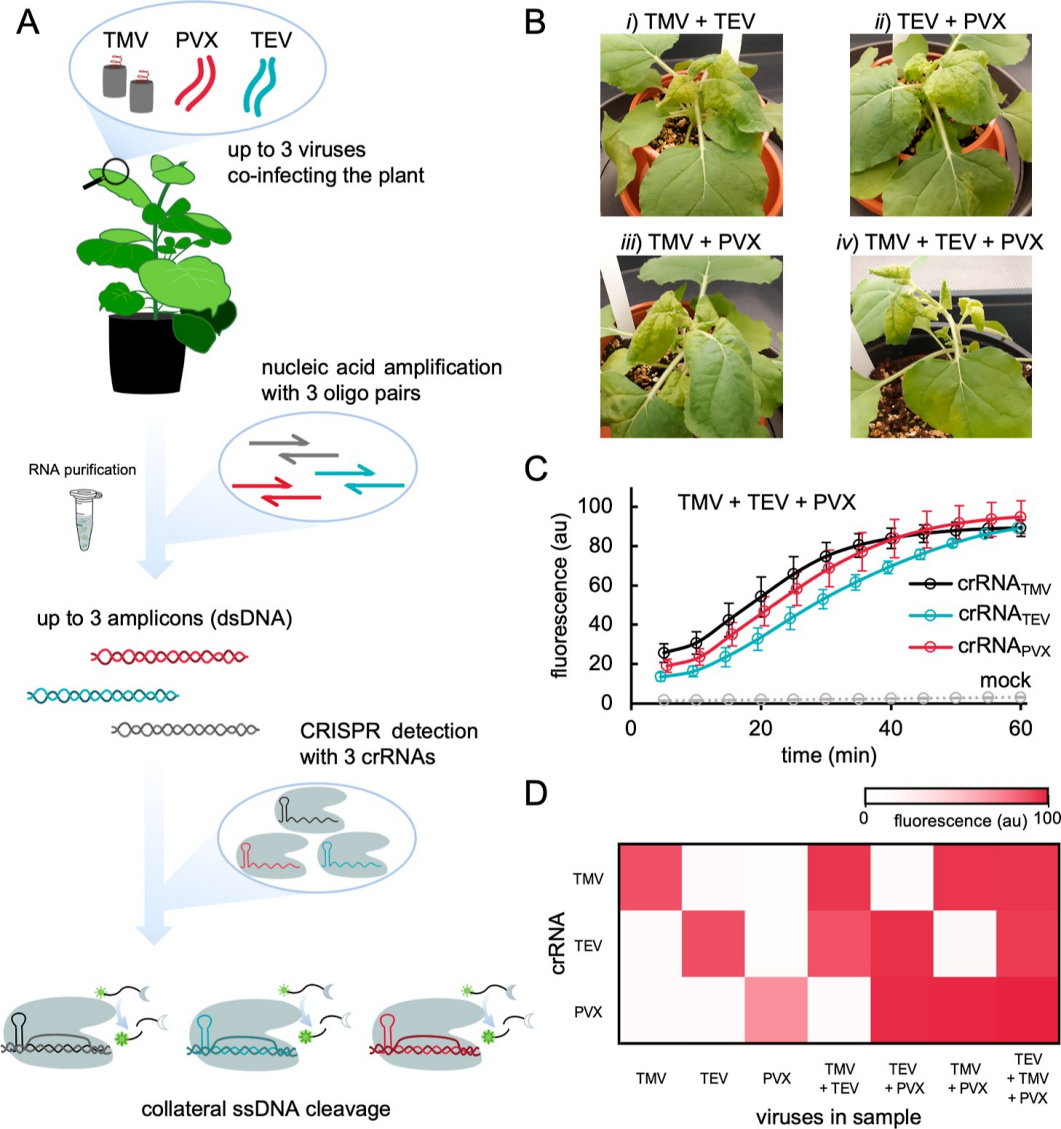
Multiplexed plant virus diagnostics with CRISPR-Cas12a. (A) Schematic representation of CRISPR-Cas12a-based detection by fluorescence in which multiple viruses infect the same plant and multiple crRNAs are deployed. (B) Representative images of *N. benthamiana* plants at 7 dpi co-infected with (i) TMV + TEV, (ii) TEV + PVX, (iii) TMV + PVX, and (iv) TMV + TEV + PVX. (C) Time-course fluorescence analysis in plants co-infected with the three viruses. Error bars represent standard deviations (*n* = 3, three CRISPR-Cas12a reactions from one amplification). (D) Heatmap of the end-point fluorescence at 1 h measured in samples amplified by RT-PCR of plant material infected with TMV, TEV, and PVX in a combinatorial way (single, double, and triple infections) obtained with Excel using the conditional formatting tool. Each crRNA is present in a specific detection reaction (*n* = 2, two CRISPR-Cas12a reactions from one amplification).

After assessing the efficient detection of single viral infections, we aimed to selectively identify the different plant viruses in complex mixtures using CRISPR-Cas12a-based detection (i.e., multiplexed diagnostics). We aimed to perform a multiplexed amplification of the different viruses infecting the plant followed by a parallelized detection with CRISPR-Cas12a. The amplification process was carried out using a set of primer pairs specific to each virus (in this case, three primer pairs). Different amplicons were generated according to the infection state of the plant from a unique sample. Infection by one virus generated one amplicon, whereas infection by three viruses generated three different amplicons. Subsequently, CRISPR-Cas12a reactions running in parallel (i.e., one reaction for each crRNA) were used to gain information about the presence of specific viruses in the sample (Figure 2A).

The analysis was successful in *N. benthamiana* plants co-infected with two (all combinations) or three viruses, using RT-PCR for amplification (Figure 2B). Figure 2C shows the three kinetic curves of ssDNA cleavage by the corresponding active crRNA-Cas12a-dsDNA complexes in the case of triple infection (reactions done separately; Figure S6 shows the kinetic curves for all virus combinations). These kinetic curves are comparable to those obtained in the case of single infections, indicating that the amplicons were properly generated. Results of multiplexed diagnostics are represented as a heatmap (Figure 2D). We roughly found the same maximal fluorescence signal at 1 h of the CRISPR-Cas12a reaction with each crRNA and each infection state. However, with the crRNA to detect PVX, we observed a higher fluorescence signal when PVX coinfected the plant with TMV or TEV than when it was alone, suggesting that PVX accumulates more in a scenario of mixed infection. This indeed agrees with previous results revealing a synergistic effect between PVX and potyviruses in plants, characterized by an increase in symptom severity.^26^ We also observed that with the crRNA to detect TMV, the fastest response is obtained (Figure S6), suggesting that the CRISPR-Cas12a reaction in that case is very efficient. The multiplexed detection of the three viruses was also performed using RT-RPA for amplification (Figure S7). Comparable results were obtained; although, as noticed before, more background signal was observed. Importantly, these results encourage the use of novel methods for multiplexed diagnostics based on CRISPR-Cas systems in molecular plant pathology.

### Field-Deployable Detection of Plant Viruses with CRISPR-Cas12a

To achieve a fast and transportable detection of plant viruses, there is a need for systems that can run without heavy equipment and do not require well-trained personnel. CRISPR-Cas systems fulfill these requirements. The ssDNA reporter can be appropriately labeled to exploit immunochromatographic assays that run in commercial lateral flow strips, thereby bypassing the need for a fluorometer to obtain the readout. In addition, the use of isothermal amplification methods such as RT-RPA, which can run at a constant and low temperature, eliminates the requirement for a thermocycler for amplification, expensive reagents, and highly skilled personnel. Here, we also used an alkaline lysis solution in which a small piece of plant tissue was submerged to generate suitable extracts for subsequent analysis (note that these extracts contain the total nucleic acids, both DNA and RNA, of the plant cells). Compared to the standard approach for RNA purification, which requires precise equipment and reagents and is time-consuming (90 min), the alkaline lysis solution allows us to obtain plant extracts in just 5 min without infrastructure.

**Figure 3:**
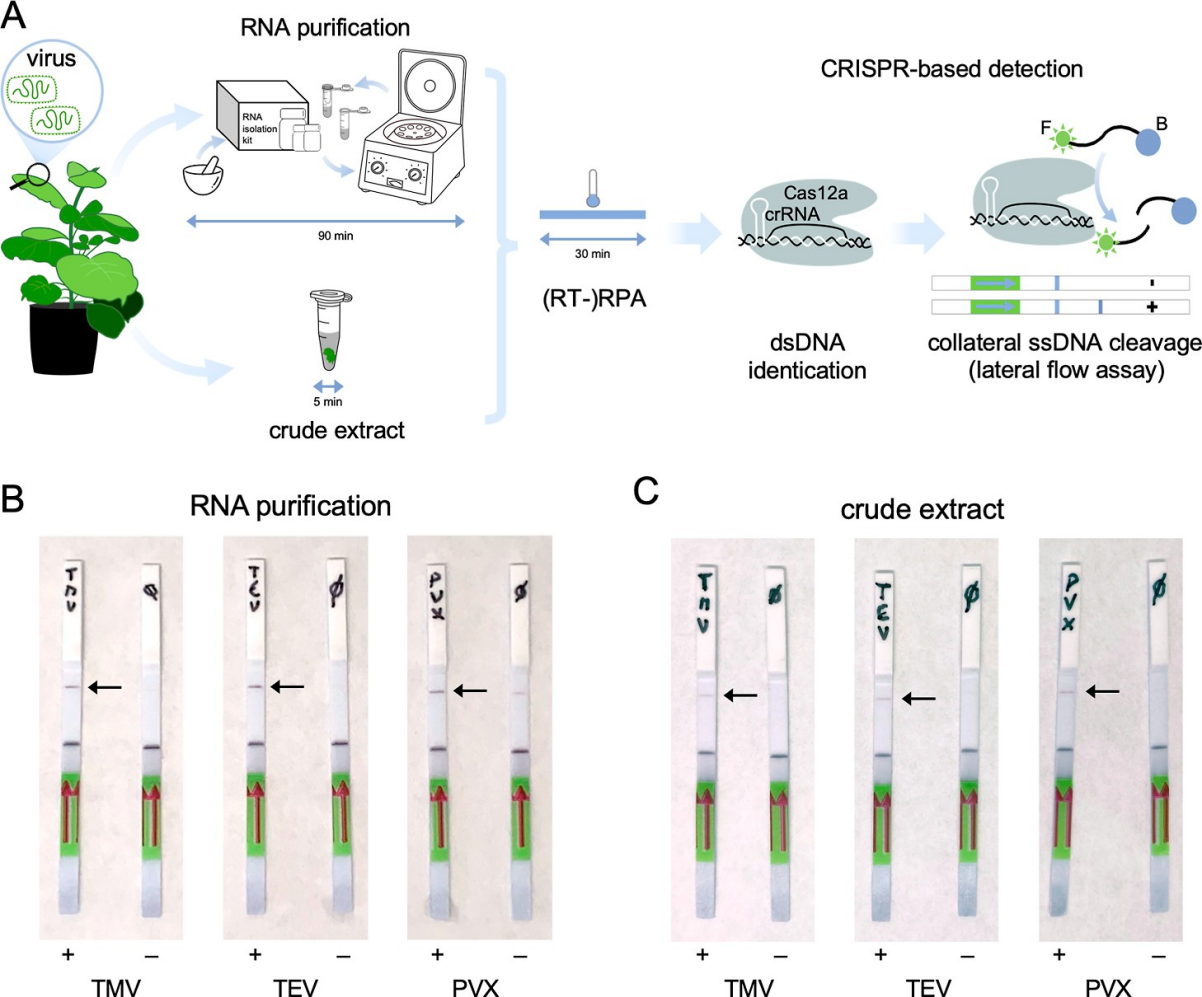
Field-deployable plant virus detection with CRISPR-Cas12a. (A) Schematic representation of CRISPR-Cas12a-based detection by a lateral flow assay. Two approaches were followed: with and without RNA purification. The ssDNA probe is labeled with a fluorophore (F, sun icon) and biotin (B, bead icon). (B,C) Representative lateral flow strips dipped into the different CRISPR-Cas12a reactions with (B) and without (C) RNA purification. Arrows point to the positive test lines in the strips.

Hence, we ran CRISPR-Cas12a reactions using an ssDNA molecule labeled with fluorescein and biotin as a probe (Figure 3A). We were able to obtain positive readouts of infection with lateral flow strips in all cases (Figure 3B,C). We performed these experiments in parallel with and without RNA purification. We noted that the test band (marked with an arrow) was less intense when RNA was not purified, suggesting as expected that the amount of viral RNA in the sample was lower. A marginal band intensity was always observed in the case of mock samples from purified RNA, especially with the crRNA to detect PVX. However, such an undesirable effect was not observed in crude extracts, which contain less free genetic material. Moreover, Figure S8 shows the fluorescence analyses (end-point and kinetic) when an alkaline lysis solution is used, which are similar to those presented before. Our results prompt the application of novel diagnostic methods to quickly recognize infections in the field and adopt the appropriate measures.

### Amplification-free Detection of Plant Viruses with CRISPR-Cas13a/d

Finally, we investigated the possibility of direct detection of viral RNA with no previous amplification step. To this end, we used the nucleases Cas13a or Cas13d, able to cleave in trans small RNA (sRNA) molecules. Three Cas13a/d-dependent crRNAs were designed to target a region coding for the coat protein, trying to avoid cross-interaction with the host transcriptome. In this case, Cas13a/d was expressed in bacteria and purified by affinity chromatography (Figure S9). *N. benthamiana* plants infected with each virus (TMV, TEV, and PVX) were used. Of note, this is a novelty introduced here, where the viral genome is directly targeted by the crRNA, rather than an amplified nucleic acid.

**Figure 4:**
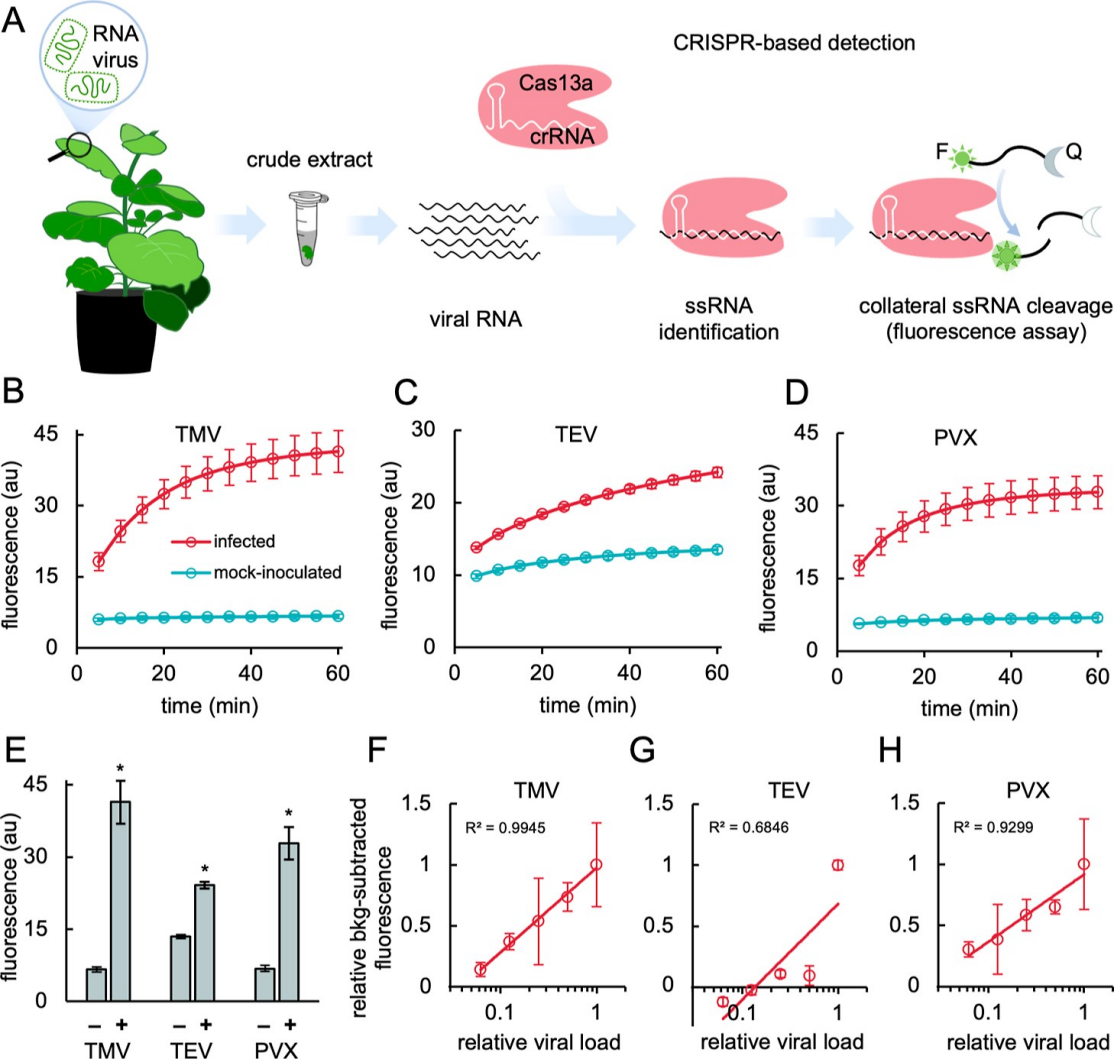
Plant virus detection with CRISPR-Cas13a. (A) Schematic representation of CRISPR-Cas13a-based detection of plant viruses by fluorescence readout. No genome amplification was done in any case. The sRNA probe is labeled with a fluorophore (F, sun icon) and a quencher (Q, moon icon). (B–D) Time-course fluorescence analysis (infected *vs* mock) in the case of TMV (B), TEV (C), or PVX (D). (E) End-point fluorescence at 1 h was measured in samples of plant material infected with TMV, TEV, PVX (+), or mock-inoculated material (−). Error bars represent standard deviations (*n* = 4, four CRISPR-Cas13a reactions with no amplification). Statistical significance (Welch’s *t*-test, two-tailed *P* < 0.05) of higher fluorescence with respect to mock-inoculated plants (*). Fold change (± standard error) was 6.21 ± 0.40 for TMV, 1.79 ± 0.04 for TEV, and 4.81 ± 0.34 for PVX. (F–H) Correlation between the relative concentration of viral RNA in the sample (proxy of viral load; gradient generated by plant extract dilutions) and the relative background-subtracted fluorescence at 30 min. The fluorescence from mock samples was considered for background subtraction. Logarithmic regression was done with Excel (*R*^2^ shown).

We ran CRISPR-Cas13a reactions to detect the viruses using a fluorogenic sRNA molecule as a probe (Figure 4A). From crude extract samples, we obtained the kinetic curves of sRNA cleavage by the active crRNA-Cas13a-RNA complex (Figure 4B–D), finding that this approach also offers good detectability (see the end-point fluorescence results shown in Figure 4E). However, compared to the CRISPR-Cas12a-based detection, the reporter was not completely processed in this case, especially with TEV. This is because the amount of viral RNA in the sample is low (at least, much lower than the number of the dsDNA amplicons). We also observed that the detection of TMV displayed the greatest difference with respect to the control for the designed crRNA set. A slight increase in fluorescence was also noticed when testing mock samples with the crRNA to detect TEV, which may be attributed to the intrinsic RNase activity of the nuclease even in the absence of a target.^27^ Moreover, our results show that a meaningful fluorescence signal to differentiate an infected plant from a noninoculated control plant can be achieved in 30 min, irrespective of the virus. Direct virus detection was also possible from purified RNA samples (Figure S10). According to the fluorescence results, the concentration of the virus in the samples is substantially higher when RNA is purified, especially in the case of TMV. With these pure samples, the basal fluorescence was also lower. In addition, we exploited the direct detection of the virus to explore the quantification of its titer in the sample (and then in the plant). From a series of diluted samples from infected plants, we found an excellent correlation between the fluorescence readout and the viral load (Figure 4F–H).

We also ran CRISPR-Cas13d reactions from purified RNA samples (Figure S11A). With respect to Cas13a, we found that Cas13d is less efficient in processing the sRNA probe, although enough to get a significant differential signal (Figure S11B–D). The difference becomes more substantial when looking at the fluorescence rate, that is, how fluorescence changes with time (Figure S11E, subplot iii). Furthermore, we were able to discriminate infected plant samples by irradiating blue light and capturing green light (Figure S11E, subplot ii), which suggests that a CRISPR-Cas13-based reaction and a portable optical device is a suitable approach for field-deployable applications. Nonetheless, we assayed an sRNA molecule labeled with fluorescein and biotin as a probe in order to use lateral flow strips, but we did not successfully obtain a distinguishable band in the test line, either with Cas13d or Cas13a. In sum, all these results with CRISPR-Cas13a/d put forward amplification-free diagnostics of plant viral infections, which represents an advancement in the field.

## Discussion

Our results indicate that nucleic acid amplification by RT-PCR or RT-RPA coupled with CRISPR-Cas12a targeting is a suitable strategy to detect RNA viruses from infected plant tissues in a simple and effective manner, extending previous work.^15−17,28^ In particular, we show that accurate detection can be performed in 40 min starting from harvested leaves at a low constant temperature of 37–40 °C, with no RNA purification by using an alkaline lysis solution and with a visual readout using lateral flow strips. The use of crude extracts for nucleic acid detection represents an ideal approach that is expected to gain momentum, provided that it is tested in widely different plants. Moreover, RPA is more rapid, sensitive, and robust than PCR but more prone to producing false positives.^8^ In this regard, the addition of the CRISPR-Cas12a step allows for more accurate detection. Other CRISPR-Cas12 systems such as CRISPR-Cas12b might also be employed.^12^ Additionally, our results with the triply infected plants demonstrate the applicability of this approach to the multiplexed diagnostics of complex mixes of plant viruses, which is a significant advance in the field. Mixed viral infections occur more frequently in nature than expected by chance;^29^ they can result in synergistic interactions among viruses that lead to devastating effects^26,30^ or, by contrast, result in antagonisms that allow the plant to better resist the infections.^30^ With CRISPR-Cas12a, such nonlinear effects may even be captured, as shown here by working with PVX.

Our results also indicate that the use of the CRISPR-Cas13a/d system constitutes a suitable strategy to directly detect RNA viruses from plant tissues, bypassing the necessity of a viral genome amplification step and obtaining a readout that scales with the viral load in the plant. Recently, this approach was used to directly detect SARS-CoV-2 in patient samples.^27^ The nuclease Cas13a was initially used to detect human viruses after an amplification process that also involved *in vitro* transcription to get RNA amplicons,^9^ and recently, this approach has also been applied to detect plant viruses.^31^ Here, we skipped the steps of RT-RPA and *in vitro* transcription thanks to the strong targeting ability and high specificity of both Cas13a and Cas13d.^18,19^ To our knowledge, this is the first demonstration of direct detection without preamplification of plant viruses with a CRISPR-Cas13 system from plant tissues.

Although many plant viruses have an RNA genome, DNA viruses also have an important economic impact on agriculture.^3^ This is the case with geminiviruses, a family of circular ssDNA viruses. LAMP amplification followed by CRISPR-Cas12a detection was already demonstrated.^32^ However, we envision that Cas12 nucleases might be used in this context to directly detect these viral genomes without amplification. Interestingly, Cas12a (able to target both dsDNA and ssDNA) or Cas12f (specifically targeting ssDNA) might be exploited to that end.^12^ In addition to viruses, organisms such as fungi, oomycetes, and bacteria can cause infectious diseases in plants.^33^ A CRISPR-Cas12 system may be used to detect the genome of these organisms as well, as already reported in some pioneer studies.^34−36^ Furthermore, because of the exquisite sequence recognition ability of these systems, the genetic diversity of a given pathogen might be detected (at least some variants). Continuous monitoring of different pathogens in the field should help to implement better control strategies incorporating ecological concepts.^37^

All in all, this work contributes to paving the way for applying CRISPR-Cas systems in modern agriculture. Specifically, our findings may aid in the development of simple, cost-effective, and rapid detection methods of plant viruses that can be employed in the field. Here, we performed diagnostics of viral infections in *N. benthamiana* and *A. thaliana* plants. Of course, this constitutes a proof of concept, so the application of these methods in crops, such as tomato, maize, or potato, is expected in the near future. Importantly, CRISPR-Cas systems are also being exploited to breed more productive and resistant plant varieties^38,39^ and as RNA interference systems to combat infections. Engineered plants with the CRISPR-Cas9 and CRISPR-Cas13 systems were shown to be resistant to DNA and RNA viruses, respectively.^40,41^ In this regard, CRISPR-Cas-based integrative programs of breeding, engineering, and diagnostics are envisioned to revolutionize agriculture and contribute to addressing the challenge of food security.

## Materials and Methods

### Plant Infection with Viruses

Five-week-old *N. benthamiana* plants were mechanically inoculated with infectious extracts of TMV, PVX, and TEV, with sequence variants MK087763.1, MT799816.1, and DQ986288 (G273A, A1119G), respectively. Frozen, 50 mg aliquots of infected tissues were ground with a ball mill (Star-Beater, VWR) and homogenized in 20 volumes of inoculation buffer [50 mM potassium phosphate (pH 8.0), 1% polyvinylpyrrolidone 10, 1% polyethylene glycol (PEG) 6000, and 10 mM 2-mercaptoethanol]. Then, a 5 μL drop of 10% carborundum in inoculation buffer was deposited on the adaxial side of a plant leaf and a cotton swab soaked in the extract containing the virus was used to spread it over the surface. In the case of multiple infections, each virus was applied in a different leaf. Plants were maintained in a growth chamber at 25 °C under a 12 h day/night photoperiod.

### Sample Preparation

Aliquots of systemic tissues (approximately 50 mg) from upper noninoculated leaves were collected at 7 days postinoculation (dpi) and frozen. RNA was purified from these aliquots using silica gel spin columns (Zymo) and eluted in 10 μL of 20 mM *tris*-HCl (pH 8.5). Different dilutions of these original samples were also prepared. Moreover, crude extracts were obtained from tissue aliquots using an alkaline PEG lysis solution as previously described,^42^ with minor modifications. In brief, leaf fragments (approximately 50 mg) were incubated with 300 μL of lysis solution (15% PEG 4000, 20 mM NaOH) for 5 min at room temperature. Tubes were vortexed and kept on ice until use. In addition, tissues of *A. thaliana* (ecotype Ei-2) infected with TEV were kindly provided by S.F. Elena (I2SysBio, Spain),^25^ and total RNA was purified following the same methodology. The infection state was confirmed by RT-PCR followed by gel electrophoresis visualization.

### Primers and crRNAs

PCR primers and RPA primers were designed in the genomic region corresponding to the coat protein of the different viruses, ensuring that a PAM sequence suitable for CRISPR-Cas12a-based detection (TTTV) was present in the amplification region at a suitable position. Primers were designed to produce amplified fragments of different sizes to allow differentiation by agarose gel electrophoresis. Primers were aligned against a drafted *N. benthamiana* genome to check off-targets (https://sefapps02.qut.edu.au).^43^ Moreover, appropriate ssDNA probes (labeled with fluorescein in the 5′ end and with a dark quencher in the 3′ end) were designed to run RT-qPCR-based detections using those primers.

The design of the Cas12a-dependent crRNAs was straightforward given the PAM position, with a spacer sequence of 20 nucleotides complementary to the target. The design of the Cas13d-dependent crRNAs was assisted by a webserver at the University of New York (https://cas13design.nygenome.org),^44^ selecting the best candidates that aligned poorly against the *N. benthamiana* transcriptome. In this case, a spacer sequence of 23 nucleotides was considered. The Cas13a-dependent crRNAs were designed using the spacers of the Cas13d-dependent crRNAs. No screening of multiple crRNAs was carried out. All crRNAs were generated by *in vitro* transcription with the TranscriptAid T7 High Yield Transcription kit (Thermo) from DNA templates (synthesized by IDT). crRNAs were then purified using the RNA Clean and Concentrator spin columns (Zymo) and quantified in a NanoDrop. Sequences are provided in Table S1.

### Viral Genome Amplification by RT-PCR

RT-PCRs were done in two steps. First, 1 μL of total RNA purified from the plant (approximately 200 ng, previously denatured at 98 °C for 1.5 min) was used for RT reactions with 500 nM of reverse primer, 1 mM dNTPs (NZYTech), 50 U RevertAid (Thermo), and 5 U RNase inhibitor (Thermo). Reactions were incubated at 42 °C for 45 min, 50 °C for 20 min, and 60 °C for 5 min, followed by an inactivation step at 70 °C for 15 min. Then, 2 μL of the RT product was used for PCR reactions with 200 μM dNTPs (NZYTech), 500 nM of forward and reverse primers (sequences provided in Table S1), and 0.4 U Netzyme DNA polymerase (Epica) in a volume of 40 μL. Reactions were incubated in a thermocycler (Eppendorf) at 94 °C for 2 min for denaturation, followed by 35 cycles of amplification at 94 °C for 40 s, 55 °C for 30 s, and 72 °C for 30 s. Multiplexed PCRs were performed under the same conditions with the three pairs of forward and reverse primers at 250 nM each.

### Viral Genome Amplification by RT-RPA

A TwistAmp Basic kit (TwistDX) was used. Forward and reverse primers (500 nM; sequences provided in Table S1), 500 U RevertAid (Thermo), and 50 U RNase inhibitor (Thermo) were added to 29.5 μL rehydration buffer for a total volume of 43.4 μL (adjusted with RNase-free water). The TwistAmp Basic reaction pellet was resuspended with this volume, and 21.7 μL in addition to 2 μL of total RNA purified from the plant (or 4 μL of crude plant extract without RNA purification) was used per reaction. To start the reaction, 280 mM magnesium acetate was added. Reactions were incubated at 40 °C for 5 min, then vortexed and spun, and re-incubated for another 25 min. Multiplexed RPAs were performed under the same conditions with the three pairs of forward and reverse primers at 160 nM each.

### Virus Detection by RT-qPCR

A TaqPath 1-Step RT-qPCR Master Mix, CG kit (Thermo) was used. 2 μL of total RNA (approximately 400 ng) was mixed with the forward and reverse primers (500 nM), the ssDNA probe (250 nM), and 5 μL of the Master Mix for a total volume of 20 μL (adjusted with RNase-free water). Reactions were performed in a QuantStudio 3 equipment (Thermo) with this protocol: incubation at 25 °C for 2 min for uracil-N glycosylation and 50 °C for 15 min for RT, followed by an inactivation step at 90 °C for 2 min and then 40 cycles of amplification at 90 °C for 3 s and 60 °C for 30 s.

### Cas13a/d Expression and Purification

Complementary DNAs of Cas13a from *L. buccalis* and Cas13d from *R. flavefaciens* were amplified by PCR using the Phusion DNA polymerase from plasmids pC0072 (Addgene #115267)^45^ and pXR001 (Addgene #109049),^19^ respectively, and inserted into the pESt expression vector by Gibson assembly. The resulting constructs included a carboxy-terminal Twin-Strep tag (TST; IBA) and were used to transform *Escherichia coli* (*E. coli*) Rosetta 2(DE3)pLysS. A 50 mL culture with lysogeny broth medium was grown overnight at 37 °C and 225 rpm. An aliquot of this culture was used to inoculate 250 mL of terrific broth medium to an OD_600_ of 0.1, which was grown under the same conditions. When the culture reached an OD_600_ of 0.6, isopropyl β-D-1-thiogalactopyranoside (IPTG) was added to a final concentration of 0.4 mM to induce protein expression, and the culture was incubated for an additional 3 h at 28 °C and 225 rpm. Bacteria were harvested and the culture medium was removed by washing the cells once with water by centrifugation. Cells were finally resuspended in 7.5 mL of water supplemented with a cocktail of protease inhibitors (complete, Roche) and stored frozen at −20 °C.

Prior to protein purification, frozen cells were thawed and incubated in lysis buffer [100 mM *tris*-HCl, 1% Nonidet P-40, 1 mM ethylenediaminetetraacetic acid (EDTA), 10 mM dithiothreitol (DTT), 12.5 U/mL benzonase, and 1 mg/mL lysozyme] at 4 °C for 45 min. Then, 150 mM KCl and 5 mM MgCl_2_ salts were added. The lysate was then clarified by centrifugation for 30 min at 85,000 g and 4 °C. The supernatant was finally filtered (0.45 μm) and loaded onto a 1 mL Strep-Tactin XT Superflow column (IBA) using an ÄKTA Prime Plus liquid chromatography system (GE) operated at 4 °C with a 1 mL/min flow rate. The column was previously equilibrated in 10 mL of chromatography buffer (100 mM *tris*-HCl, 1% Nonidet P-40, 1 mM EDTA, 10 mM DTT, 150 mM KCl, and 5 mM MgCl_2_) before loading the extract. The column was then washed and Cas13d-TST eluted with 20 mL of chromatography buffer supplemented with 50 mM D-biotin. 1 mL aliquots were collected during the elution step.

Cas13a/d-TST was analyzed in the *E. coli* extract and chromatography-eluted fractions by western blot after separation by sodium dodecyl sulfate-polyacrylamide gel electrophoresis (PAGE) in 10% gels, using an anti-TST monoclonal antibody conjugated to horseradish peroxidase (IBA). Quantification of protein concentration was done by a Bradford assay.

### CRISPR-Cas12a Reaction

A mix of 50 nM Cas12a from *L. bacterium* (NEB) and 62.5 nM crRNA was incubated for 30 min in NEBuffer 2.1 [10 mM tris-HCl, 50 mM NaCl, 10 mM MgCl_2_, and 100 μg/mL BSA (pH 7.9); NEB] at room temperature. In a 96-well plate (MicroAmp Fast, Applied), 2 μL of amplified DNA was mixed with 17 μL of the CRISPR-Cas12a ribonucleoprotein preparation and 500 nM of the ssDNA probe (TTATT, labeled with fluorescein-quencher at the ends) to make a total volume of 20 μL. Incubating for 1 h at 37 °C, green fluorescence was measured every 5 min in a real-time PCR machine (QuantStudio 3, Thermo). Excitation was at 470/15 nm, and emission was at 520/15 nm. In the case of multiplexed detection, each crRNA was present in a separate well, and then, different CRISPR reactions were run in parallel on the same plate. Quantitative values are provided in Table S2.

### CRISPR-Cas13a/d Reaction

A mix of 10 nM Cas13a (or 100 nM Cas13d) and 62.5 nM crRNA was incubated for 30 min at room temperature in NEBuffer 2.1. In a 96-well plate (MicroAmp Fast, Applied), 2 μL of total RNA purified from the plant (or 3 μL of crude plant extract without RNA purification) was mixed with 17 μL of the CRISPR-Cas13a/d ribonucleoprotein preparation and 500 nM of the RNA probe (UUUUU, labeled with fluorescein-quencher) to make a total volume of 20 μL. Incubating for 2 h at 37 °C, green fluorescence was measured every 10 min in a real-time PCR machine (QuantStudio 3, Thermo). Quantitative values are provided in Table S2.

### Lateral Flow Assay

CRISPR-Cas12a reactions were prepared in the same manner as in the fluorescence assay and incubated for 2 h at 37 °C, in this case with the ssDNA probe TTATT labeled with fluorescein-biotin at the ends and at 200 nM. Then, 80 μL GenLine Dipstick buffer (Milenia) supplemented with 5% PEG 6000 was added for 1:5 dilution. The lateral flow strip (HybriDetect, Milenia) was dipped into the reaction tube and images were captured with a smartphone after 5 min.

### Fluorescence Imaging

CRISPR-Cas13d reaction tubes were irradiated with blue light and images were acquired with a 2.8-Mpixel camera with a filter for green fluorescence in a light microscope (Leica MSV269). Using the commercial software provided by Leica, the contrast of the images was adjusted to enhance the visualization of the differential fluorescence among samples.

## Supporting Information Available

Genomic architectures of the plant viruses, detection by RT-qPCR, additional results of Cas12a-based detection, nuclease expression and purification scheme, additional results of Cas13a/d-based detection, nucleic acid sequences, and numerical data (PDF)
