# Supplementary material for: Diagnostics of Infections Produced by the Plant Viruses TMV, TEV, and PVX with CRISPR-Cas12 and CRISPR-Cas13
Source: ACS Synth Biol. 2022 Jul 6;11(7):2384–93. doi: 10.1021/acssynbio.2c00090 (PMC9295153; doi:10.1021/acssynbio.2c00090)
Supplement: Supplementary file 1 — sb2c00090_si_001.pdf [file sb2c00090_si_001.pdf]

Supporting Information

Diagnostics of infections produced by the plant viruses TMV, TEV, and PVX with CRISPR-Cas12 and CRISPR-Cas13

María-Carmen Marqués[1,†], Javier Sánchez-Vicente[2,†], Raúl Ruiz[1,†], Roser Montagud-Martínez[1,†], Rosa Márquez-Costa[1], Gustavo Gómez[1], Alberto Carbonell[2], José-Antonio Daròs[2,*], and Guillermo Rodrigo[1,*]

[1]Institute for Integrative Systems Biology (I2SysBio), CSIC – Universitat de València, 46980 Paterna, Spain

[2]Instituto de Biología Molecular y Celular de Plantas, CSIC – Universitat Politècnica de València, 46022 València, Spain

[†]Equal contribution to this work

[*]Correspondence: jadaros@ibmcp.upv.es, guillermo.rodrigo@csic.es

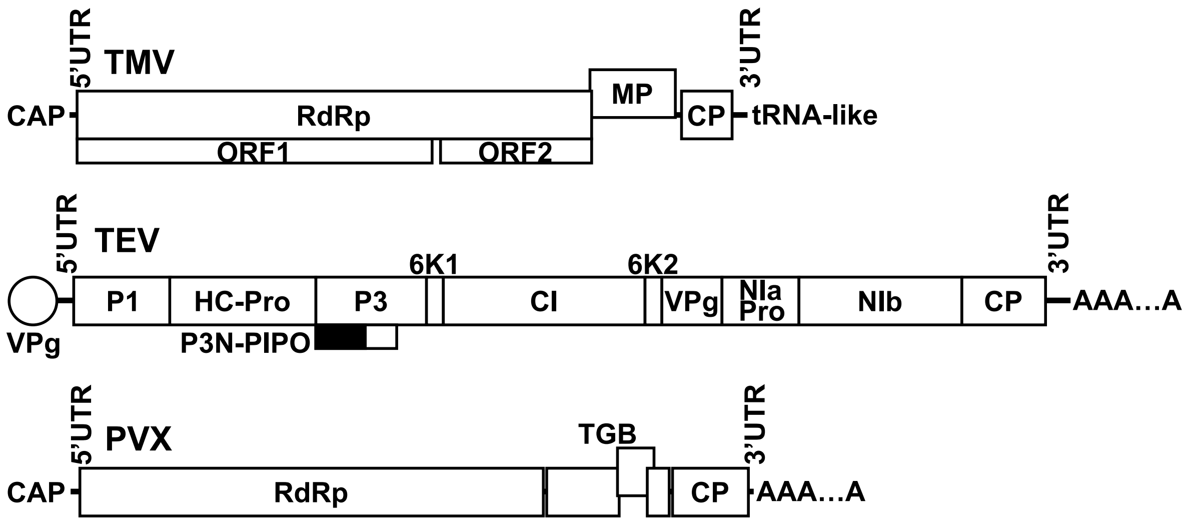

Figure S1: Genomic architectures of the plant viruses considered in this work. TMV is a *Tobamovirus* with an RNA genome of ~6.4 kb that deploys 4 proteins. TEV is a *Potyvirus* with an RNA genome of ~9.5 kb that deploys 11 proteins. PVX is a *Potexvirus* with an RNA genome of ~6.4 kb that deploys 5 proteins.

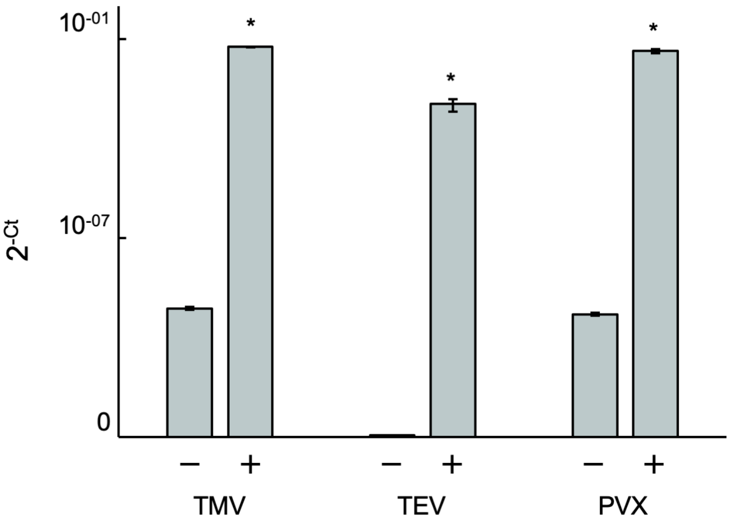

Figure S2: Plant virus detection by RT-qPCR. The value of the negative power to 2 of the cycle threshold (Ct) obtained is represented. Error bars correspond to standard deviations (n = 3). *Statistical significance (Welch's t-test, two-tailed $P < 0.05$). The fold change (± standard error) is $7.68 \cdot 10^7$ (± $0.43 \cdot 10^7$) for TMV, ∞ for TEV (*i.e.*, no Ct value in the case of no infection), and $8.78 \cdot 10^7$ (± $0.82 \cdot 10^7$) for PVX.

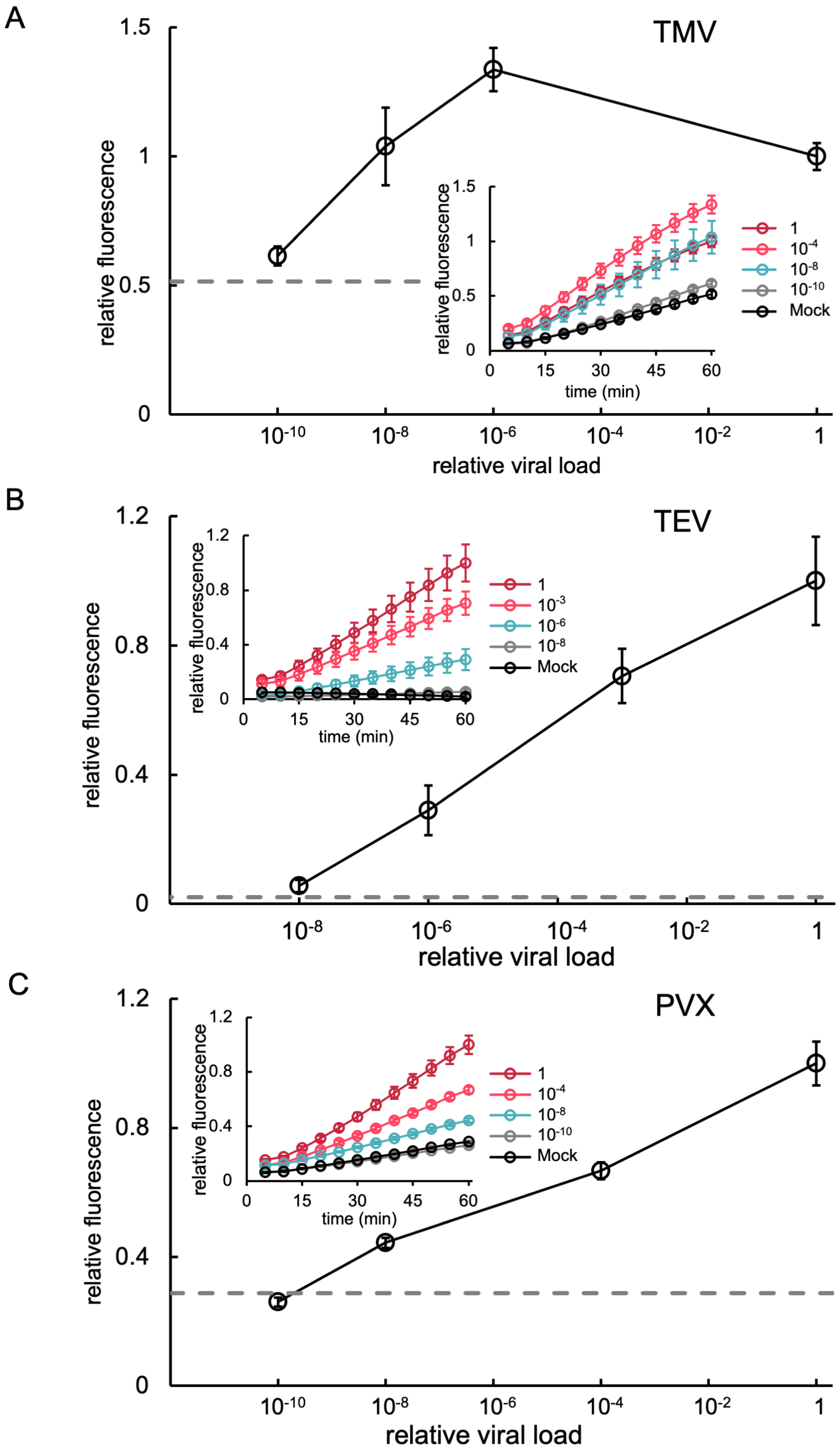

Figure S3: Plant virus detection with CRISPR-Cas12a for different viral loads. End-point relative fluorescence measured from different diluted samples infected with A) TMV, B) TEV, or C) PVX at 1 h (normalized to the case of relative viral load = 1). Amplification performed by RT-PCR. The *x* axis represents a relative viral load calculated from the dilution factor

applied to the original sample (*i.e.*, a relative viral load of 10^{-4} means that the original sample was diluted 10^4 times before performing the RT-PCR and CRISPR-Cas12a reaction). This simulates samples in which the virus is present at different concentrations (eventually, due to shorter collection times or lower viral fitness). The horizontal dashed line corresponds to the fluorescence level obtained with a mock sample (*i.e.*, no virus). The insets show the kinetic assays of viral detection with CRISPR-Cas12a.

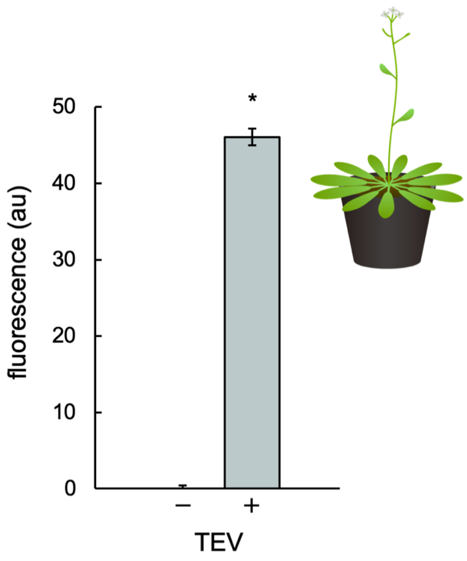

Figure S4: TEV detection with CRIPSR-Cas12a in *Arabidopsis thaliana*. Amplification performed by RT-PCR. Error bars correspond to standard deviations (n = 3). *Statistical significance (Welch's t-test, two-tailed P < 0.05). The fold change (± standard error) is 1336 (± 1089).

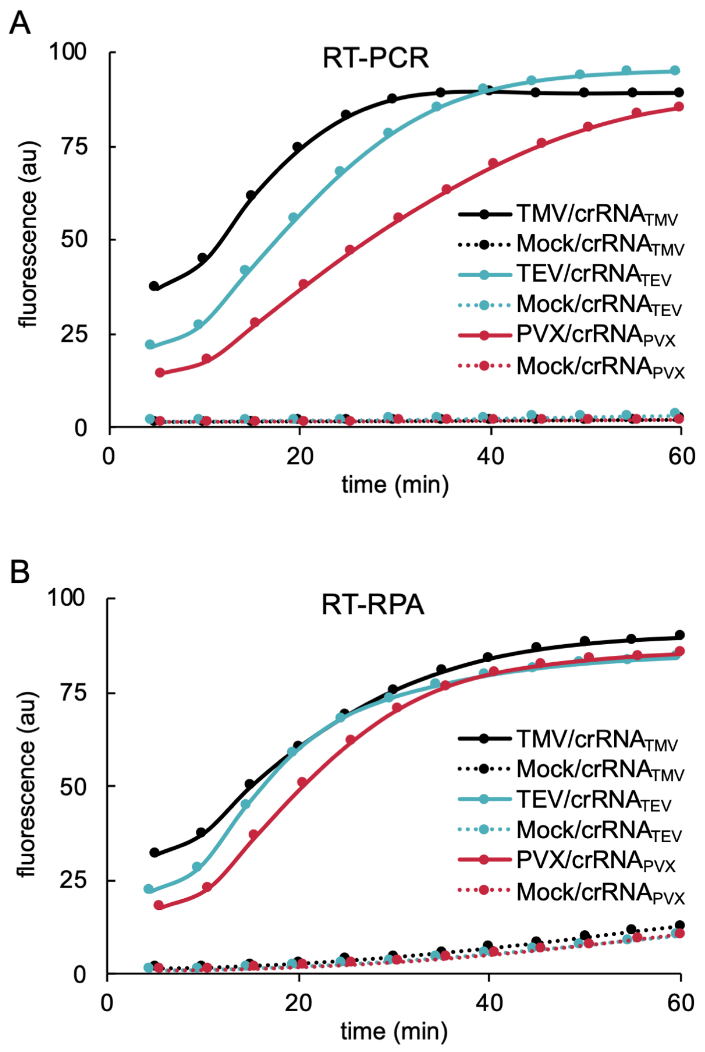

Figure S5: Kinetic assay of CRISPR-Cas12a-based detection of DNA amplicons from viral RNA. A) Amplification by RT-PCR or B) by RT-RPA. Each line corresponds to a representative replicate. Solid lines correspond to infected plants, while dotted lines to healthy plants.

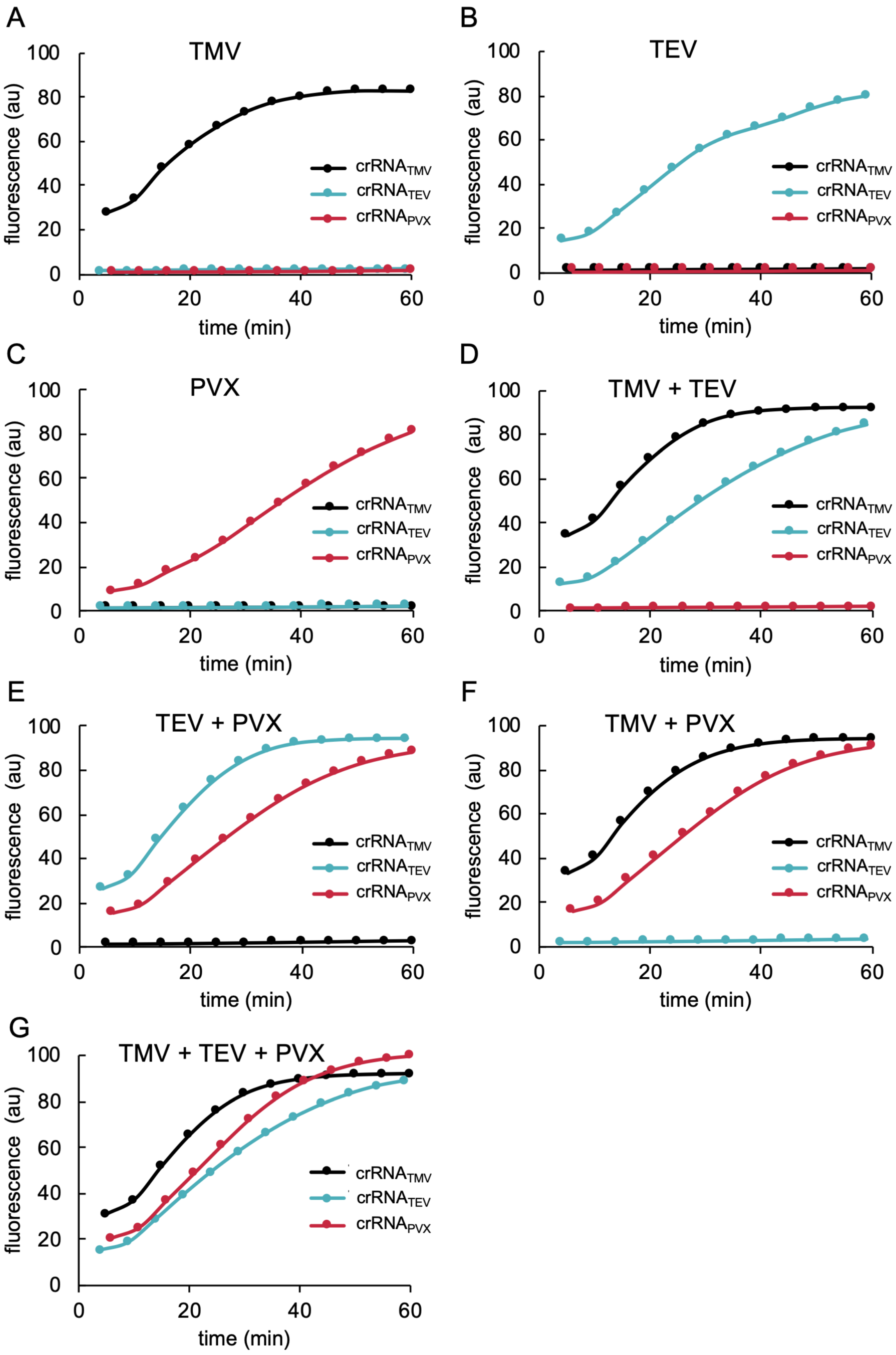

Figure S6: Kinetic assay of CRISPR-Cas12a-based multiplexed detection of DNA amplicons from viral RNAs. In all cases, amplification was done by RT-PCR. Each line corresponds to a representative replicate. A) Plants infected with TMV. B) Plants infected

with TEV. C) Plants infected with PVX. D) Plants co-infected with TMV and TEV. E) Plants co-infected with TEV and PVX. F) Plants co-infected with TMV and PVX. G) Plants co-infected with TMV, TEV, and PVX.

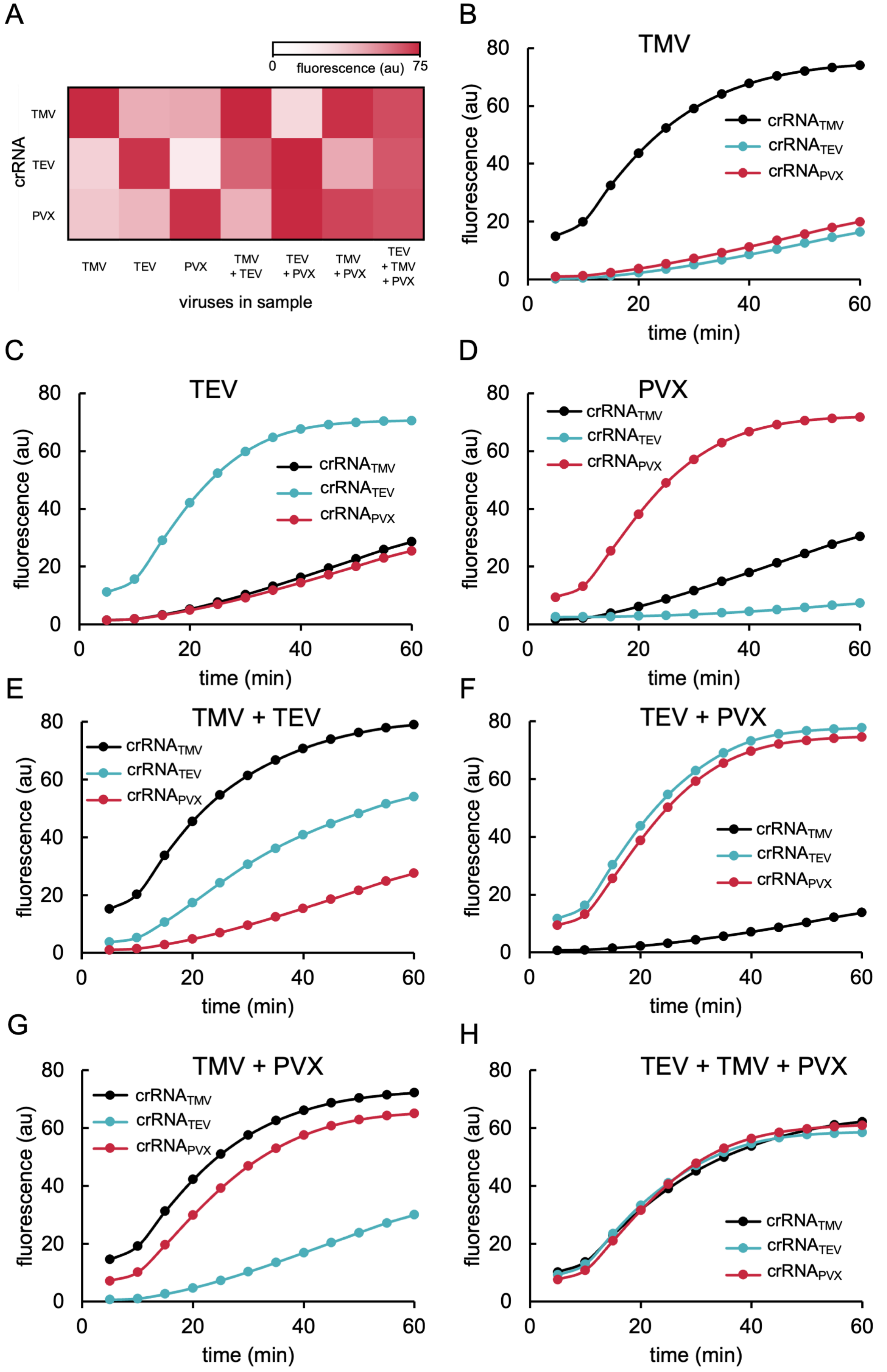

Figure S7: Multiplexed plant virus diagnostics with CRISPR-Cas12a using RT-RPA for amplification. A) Heatmap of the end-point fluorescence at 1 h measured in samples amplified by RT-RPA of plant material infected with TMV, TEV, and PVX in a combinatorial way (single, double, and triple infections). Done with Excel using the conditional formatting tool. Each crRNA is present in a specific detection reaction (*n* = 2, two CRISPR-Cas12a reactions from one amplification). B-H) Kinetic assay of CRISPR-Cas12a-based multiplexed detection of DNA amplicons from viral RNAs. Each line corresponds to a representative replicate. B) Plants infected with TMV. C) Plants infected with TEV. D) Plants infected with PVX. E) Plants co-infected with TMV and TEV. F) Plants co-infected with TEV and PVX. G) Plants co-infected with TMV and PVX. H) Plants co-infected with TMV, TEV, and PVX.

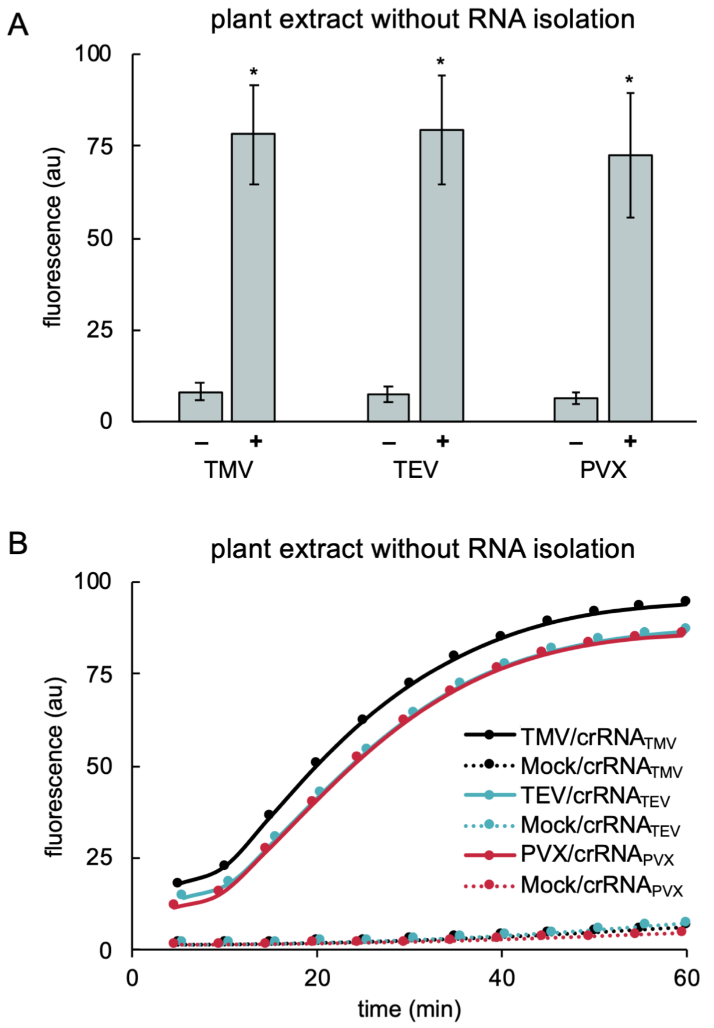

Figure S8: CRISPR-Cas12a-based viral detection from plant extract without RNA isolation. A) End-point fluorescence measured from samples infected with TMV, TEV, or PVX (+) compared to mock inoculated plants (–) at 1 h. Error bars correspond to standard deviations (n = 4). *Statistical significance (Welch's t-test, two-tailed P < 0.05). The fold change (± standard error) is 9.78 (± 1.69) for TMV, 10.52 (± 1.74) for TEV, and 11.22 (± 2.03) for PVX. B) Kinetic assay of viral detection using plant extract without RNA isolation and CRISPR-Cas12a; previous amplification by RT-RPA. Each line corresponds to a representative replicate. Solid lines correspond to infected plants, while dotted lines to healthy plants.

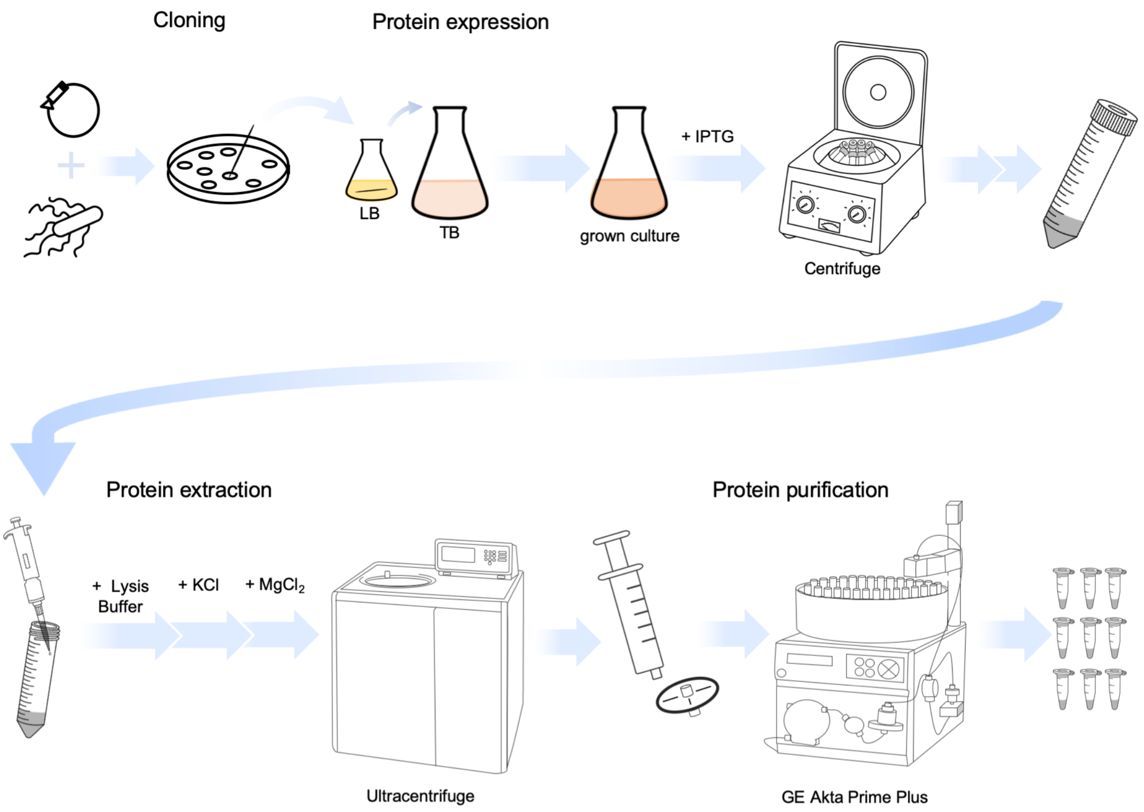

Figure S9: Experimental scheme for expressing (in *E. coli*) and purifying (by HPLC) the nucleases Cas13a or Cas13d.

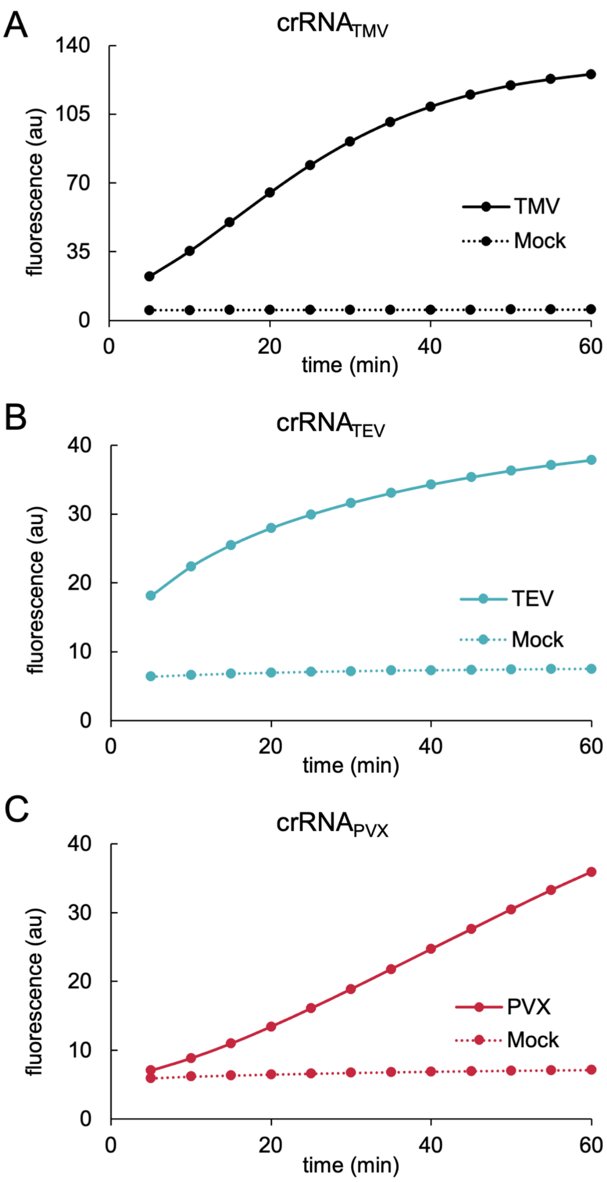

Figure S10: Plant virus detection with CRISPR-Cas13a from purified RNA. Time-course fluorescence analysis (infected *vs*. mock) in the case of TMV (A), TEV (B), or PVX (C). Each line corresponds to a representative replicate.

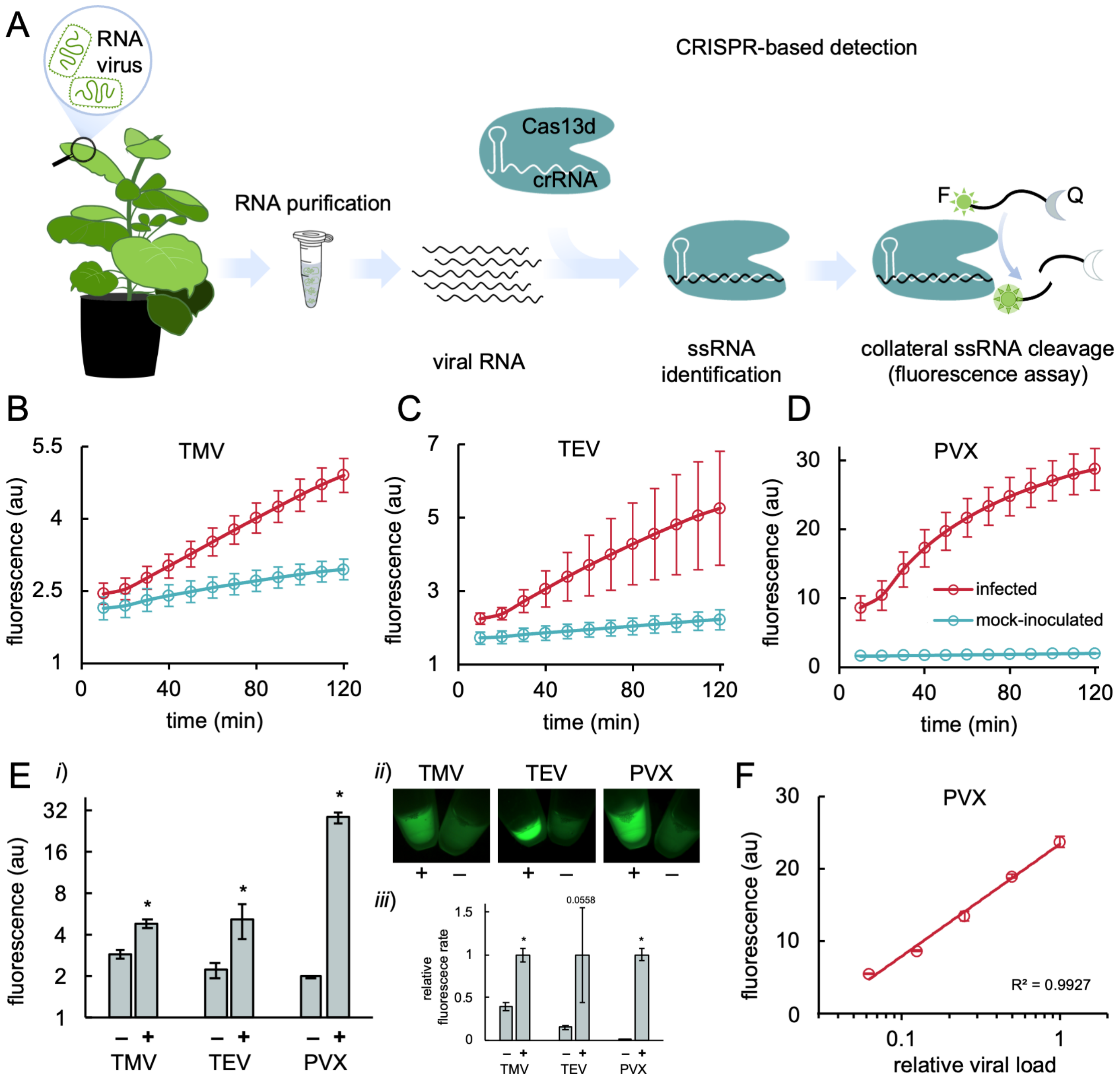

Figure S11: Plant virus detection with CRISPR-Cas13d from purified RNA. A) Schematic representation of CRISPR-Cas13d-based detection of plant viruses by fluorescence readout. No genome amplification was done in any case. The sRNA probe is labelled with a fluorophore (F, sun icon) and a quencher (Q, moon icon). B - D) Time-course fluorescence analysis (infected *vs*. mock) in the case of TMV (B), TEV (C), or PVX (D). E) *i*) End-point fluorescence at 2 h measured in samples of plant material infected with TMV, TEV, or PVX (+) or mock-inoculated material (–). On the right, *ii*) fluorescence images of the reaction tubes are shown, as well as *iii*) a subplot with the relative fluorescence rate (slope, t < 1 h; normalized to the infection state). Error bars represent standard deviations

(*n* = 4, four CRISPR-Cas13d reactions with no amplification). Statistical significance (Welch's *t*-test, two-tailed $P < 0.05$) of higher fluorescence with respect to mock-inoculated plants (*). In (E, *i*), fold change (± standard error) of 1.67 ± 0.09 for TMV, 2.37 ± 0.38 for TEV, and 14.41 ± 0.78 for PVX. In (E, *iii*), fold change (± standard error) of 2.53 ± 0.19 for TMV, 6.60 ± 1.92 for TEV, and 87.16 ± 5.25 for PVX. F) Correlation between the relative concentration of viral RNA in the sample (proxy of viral load; gradient generated by plant extract dilutions) and fluorescence at 30 min (*n* = 3, three CRISPR-Cas13d reactions from a pooled sample). Logarithmic regression done with Excel (R^2 shown).

Table S1: cDNA sequences of crRNAs and primers used in this work.

Description	Sequence
Cas12a crRNA to detect TMV	TAATTTCTACTAAGTGTAGATAGGTGTACAGGTACAATGCG
Cas13d crRNA to detect TMV	CAAGTAAACCCCTACCAACTGGTCGGGGTTTGAAACTACA CCTTAAAGTCACTGTCAGG
Cas13a crRNA to detect TMV	GGACCACCCCAAAAATGAAGGGGACTAAAAC TACACCTTAAAGTCACTGTCAGG
Forward primer to amplify TMV by PCR	AGACAATTCAGTGAGGTGTGG
Reverse primer to amplify TMV by PCR	CCTAACAGTGCTGTGACTAGC
Forward primer to amplify TMV by RPA	GACAATTCAGTGAGGTGTGGAAACCTTCAC
Reverse primer to amplify TMV by RPA	TCGAATGCACCTAACAGTGCTGTGACTAGC
Cas12a crRNA to detect TEV	TAATTTCTACTAAGTGTAGATATACCCGCTGAAACCAATGG
Cas13d crRNA to detect TEV	CAAGTAAACCCCTACCAACTGGTCGGGGTTTGAAACGTCC CATTTTCTATGCACCACAC
Cas13a crRNA to detect TEV	GGACCACCCCAAAAATGAAGGGGACTAAAAC GTCCCATTTTCTATGCACCACAC
Forward primer to amplify TEV by PCR	GTTATGATGGATGGTGAGGAGC
Reverse primer to amplify TEV by PCR	TGTATGGTCGCTCCCTATTCC
Forward primer to amplify TEV by RPA	GAACTTGGGTTATGATGGATGGTGAGGAGC
Reverse primer to	ACCTAGGCATGTATGGTCGCTCCCTATTCC

amplify TEV by RPA	
Cas12a crRNA to detect PVX	TAATTTCTACTAAGTGTAGATAAGCCTGAGCACAAATTCGC
Cas13d crRNA to detect PVX	CAAGTAAACCCCTACCAACTGGTCGGGGTTTGAAACAAATTGCCTAAGTGTGCACACCT
Cas13a crRNA to detect PVX	GGACCACCCCAAAAATGAAGGGGACTAAAACAAATTGCCTAAGTGTGCACACCT
Forward primer to amplify PVX by PCR	CAACAGTCCACCTGCTAACTGG
Reverse primer to amplify PVX by PCR	CAGCAGTTTGGGCAGCATTC
Forward primer to amplify PVX by RPA	ACAACAGTCCACCTGCTAACTGGCAAGCAC
Reverse primer to amplify PVX by RPA	AGCAGTTTGGGCAGCATTCATTTCAGCTTC
qPCR probe for TMV	/56-FAM/AGGTTCCCTGACAGTGACTTTAAGGTGT/3IABkFQ/
qPCR probe for TEV	/56-FAM/CGCAGCCAACACTGAGGCAAATTA/3IABkFQ/
qPCR probe for PVX	/56-FAM/TTCAATGGAGTCACCAACCCAGCT/3IABkFQ/

Table S2: Experimental result values.

Figure	Condition	Mean (AU)	SD (AU)
1E	TMV -	3.91	3.79
1E	TMV +	84.01	12.18
1E	TEV -	5.06	3.78
1E	TEV +	81.67	11.67
1E	PVX -	2.04	0.32
1E	PVX +	82.55	4.84
1F	TMV -	15.21	2.57
1F	TMV +	71.00	5.12

1F	TEV -	10.68	3.06
1F	TEV +	80.01	5.81
1F	PVX -	20.80	12.61
1F	PVX +	83.43	8.03
2C	$crRNA_{TMV}$	89.24	4.22
2C	$crRNA_{TEV}$	89.13	0.25
2C	$crRNA_{PVX}$	94.88	8.37
2D	TMV $crRNA_{TMV}$	80.43	
2D	TMV $crRNA_{TEV}$	2.08	
2D	TMV $crRNA_{PVX}$	2.03	
2D	TEV $crRNA_{TMV}$	2.49	
2D	TEV $crRNA_{TEV}$	81.57	
2D	TEV $crRNA_{PVX}$	2.46	
2D	PVX $crRNA_{TMV}$	1.80	
2D	PVX $crRNA_{TEV}$	1.65	
2D	PVX $crRNA_{PVX}$	52.25	
2D	TMV + TEV $crRNA_{TMV}$	91.83	
2D	TMV + TEV $crRNA_{TEV}$	79.81	
2D	TMV + TEV $crRNA_{PVX}$	2.49	
2D	TEV + PVX $crRNA_{TMV}$	2.42	
2D	TEV + PVX $crRNA_{TEV}$	94.74	
2D	TEV + PVX $crRNA_{PVX}$	96.85	
2D	TMV + PVX $crRNA_{TMV}$	92.72	
2D	TMV + PVX $crRNA_{TEV}$	3.66	
2D	TMV + PVX $crRNA_{PVX}$	97.78	
2D	TMV + TEV + PVX $crRNA_{TMV}$	91.68	
2D	TMV + TEV + PVX $crRNA_{TEV}$	89.27	
2D	TMV + TEV + PVX $crRNA_{PVX}$	99.71	
4B	mock-inoculated 60 min	41.39	4.43
4B	infected 60 min	6.67	0.49
4C	mock-inoculated 60 min	24.17	0.69
4C	infected 60 min	13.48	0.46
4D	mock-inoculated 60 min	32.85	3.41
4D	infected 60 min	6.83	0.66

4F	TMV 1	1	0.34
4F	TMV 0.5	0.74	0.12
4F	TMV 0.25	0.54	0.35
4F	TMV 0.125	0.37	0.07
4F	TMV 0.063	0.14	0.06
4G	TEV 1	1	0.04
4G	TEV 0.5	0.09	0.08
4G	TEV 0.25	0.11	0.03
4G	TEV 0.125	-0.02	0.04
4G	TEV 0.063	-0.12	0.03
4H	PVX 1	1	0.37
4H	PVX 0.5	0.75	0.06
4H	PVX 0.25	0.58	0.13
4H	PVX 0.125	0.39	0.29
4H	PVX 0.063	0.30	0.06
S2	TMV -	7.54E-10	6.96E-11
S2	TMV +	5.79E-02	1.63E-03
S2	TEV -	N/A	N/A
S2	TEV +	1.11E-03	4.48E-04
S2	PVX -	5.00E-10	4.31E-11
S2	PVX +	4.39E-02	6.00E-03
S3	TMV 1 60 min	46.00	2.40
S3	TMV 1E-06 60 min	61.47	3.85
S3	TMV 1E-08 60 min	47.76	6.92
S3	TMV 1E-10 60 min	28.25	1.72
S3	TMV Mock 60 min	23.64	1.50
S3	TEV 1 60 min	22.82	3.11
S3	TEV 1E-03 60 min	16.11	1.93
S3	TEV 1E-06 60 min	6.61	1.76
S3	TEV 1E-8 60 min	1.28	0.42
S3	TEV Mock 60 min	0.48	0.41
S3	PVX 1 60 min	8.30	0.56
S3	PVX 1E-06 60 min	5.54	0.22
S3	PVX 1E-08 60 min	3.68	0.13

S3	PVX 1E-10 60 min	2.16	0.11
S3	PVX Mock 60 min	2.39	0.06
S4	TEV -	0.03	0.04
S4	TEV +	46.03	1.09
S5A	TMV/crRNA$_{TMV}$ 60 min	88.87	
S5A	Mock/crRNA$_{TMV}$ 60 min	2.04	
S5A	TEV/crRNA$_{TEV}$ 60 min	94.83	
S5A	Mock/crRNA$_{TEV}$ 60 min	3.11	
S5A	PVX/crRNA$_{PVX}$ 60 min	85.19	
S5A	Mock/crRNA$_{PVX}$ 60 min	1.81	
S5B	TMV/crRNA$_{TMV}$ 60 min	89.60	
S5B	Mock/crRNA$_{TMV}$ 60 min	12.61	
S5B	TEV/crRNA$_{TEV}$ 60 min	84.22	
S5B	Mock/crRNA$_{TEV}$ 60 min	9.18	
S5B	PVX/crRNA$_{PVX}$ 60 min	85.30	
S5B	Mock/crRNA$_{PVX}$ 60 min	9.20	
S6A	crRNA$_{TMV}$ 60 min	83.06	
S6A	crRNA$_{TEV}$ 60 min	2.42	
S6A	crRNA$_{PVX}$ 60 min	1.69	
S6B	crRNA$_{TMV}$ 60 min	1.80	
S6B	crRNA$_{TEV}$ 60 min	79.69	
S6B	crRNA$_{PVX}$ 60 min	1.40	
S6C	crRNA$_{TMV}$ 60 min	2.05	
S6C	crRNA$_{TEV}$ 60 min	2.49	
S6C	crRNA$_{PVX}$ 60 min	81.29	
S6D	crRNA$_{TMV}$ 60 min	92.08	
S6D	crRNA$_{TEV}$ 60 min	84.40	
S6D	crRNA$_{PVX}$ 60 min	2.29	
S6E	crRNA$_{TMV}$ 60 min	2.73	
S6E	crRNA$_{TEV}$ 60 min	94.32	
S6E	crRNA$_{PVX}$ 60 min	88.51	
S6F	crRNA$_{TMV}$ 60 min	93.78	
S6F	crRNA$_{TEV}$ 60 min	3.40	
S6F	crRNA$_{PVX}$ 60 min	90.51	

S6G	crRNA$_{TMV}$ 60 min	91.93	
S6G	crRNA$_{TEV}$ 60 min	88.85	
S6G	crRNA$_{PVX}$ 60 min	99.63	
S7B	crRNA$_{TMV}$ 60 min	74.04	
S7B	crRNA$_{TEV}$ 60 min	16.34	
S7B	crRNA$_{PVX}$ 60 min	19.92	
S7C	crRNA$_{TMV}$ 60 min	28.58	
S7C	crRNA$_{TEV}$ 60 min	70.53	
S7C	crRNA$_{PVX}$ 60 min	25.39	
S7D	crRNA$_{TMV}$ 60 min	30.48	
S7D	crRNA$_{TEV}$ 60 min	7.34	
S7D	crRNA$_{PVX}$ 60 min	71.73	
S7E	crRNA$_{TMV}$ 60 min	78.97	
S7E	crRNA$_{TEV}$ 60 min	54.07	
S7E	crRNA$_{PVX}$ 60 min	27.55	
S7F	crRNA$_{TMV}$ 60 min	13.71	
S7F	crRNA$_{TEV}$ 60 min	77.66	
S7F	crRNA$_{PVX}$ 60 min	74.48	
S7G	crRNA$_{TMV}$ 60 min	72.22	
S7G	crRNA$_{TEV}$ 60 min	29.95	
S7G	crRNA$_{PVX}$ 60 min	65.01	
S7H	crRNA$_{TMV}$ 60 min	58.49	
S7H	crRNA$_{TEV}$ 60 min	62.20	
S7H	crRNA$_{PVX}$ 60 min	60.95	
S8A	TMV -	7.99	2.41
S8A	TMV +	78.22	13.43
S8A	TEV -	7.54	2.07
S8A	TEV +	79.34	14.69
S8A	PVX -	6.46	1.77
S8A	PVX +	72.50	17.07
S10A	TMV - 60 min	5.55	
S10A	TMV + 60 min	125.38	
S10B	TEV - 60 min	7.50	
S10B	TEV + 60 min	37.86	

S10C	PVX - 60 min	7.13	
S10C	PVX + 60 min	35.94	
S11B	mock-inoculated 120 min	4.90	0.35
S11B	infected 120 min	2.94	0.22
S11C	mock-inoculated 120 min	5.26	1.55
S11C	infected 120 min	2.22	0.27
S11D	mock-inoculated 120 min	28.73	3.03
S11D	infected 120 min	1.99	0.04
S11E iii)	TMV -	0.40	0.05
S11E iii)	TMV +	1.00	0.08
S11E iii)	TEV -	0.15	0.02
S11E iii)	TEV +	1.00	0.56
S11E iii)	PVX -	0.01	< 0.01
S11E iii)	PVX +	1.00	0.073
